# Efficacy of flexible ureterorenoscopy with holmium laser in the management of calyceal diverticular calculi

**DOI:** 10.1007/s00240-024-01552-9

**Published:** 2024-03-30

**Authors:** Shi-Ping Zeng, Yi-Fei Sun, Han-Yang Yu, Jian Yang, Ke-Fei Deng

**Affiliations:** 1 Department of Urology, Zigong First People’s Hospital, Zigong, Sichuan China; 2 Neurological Disease Laboratory, West China Hospital of Sichuan University, Chengdu, Sichuan China; 3 Department of Urology, The People’s Hospital of Weiyuan, Neijiang, Sichuan China

**Keywords:** Flexible ureterorenoscopy, Holmium laser, Calyceal diverticula, Calyceal diverticular calculi, Stone

## Abstract

The purpose of this study was to evaluate the efficacy and safety of flexible ureteroscopy with holmium laser lithotripsy in the management of calyceal diverticular calculi. In this study, we retrospectively analyzed the clinical data of 27 patients with calyceal diverticular calculi admitted to the Department of Urology of the Zigong First People’s Hospital from May 2018 to May 2021. Intraoperatively, the diverticular neck was found in all 27 patients, but flexible ureterorenoscopy lithotripsy was not performed in 2 cases because of the slender diverticular neck, and the success rate of the operation was 92.6%. Of the 25 patients with successful lithotripsy, the mean operative time was 76.9 ± 35.5 (43–200) min. There were no serious intraoperative complications such as ureteral perforation, mucosal avulsion, or hemorrhage. Postoperative minor complications (Clavien classification I-II) occurred in 4 (16%) patients. The mean hospital stay was 4.4 ± 1.7 (3–12) days. The stone-free rate was 80% at the 1-month postoperative follow-up. After the second-stage treatment, the stone-free rate was 88%. In 22 cases with complete stone clearance, no stone recurrence was observed at 5.3 ± 2.6 (3–12) months follow-up. This retrospective study demonstrated that flexible ureterorenoscopy with holmium laser is a safe and effective choice for the treatment of calyceal diverticular calculi, because it utilizes the natural lumen of the human body and has the advantages of less trauma, fewer complications, and a higher stone-free rate.

## Introduction

Calyceal diverticula are rare malformations of the renal parenchyma, which is connected to the renal collecting system through a small channel. Narrowing of the channel often leads to diverticulum expansion, urinary stasis, stone formation, and infection. In patients undergoing intravenous pyelogram, calyceal diverticula can be seen in about 0.21–0.45% of patients [1]. About 9.5–50% of the calyceal diverticula will be combined with diverticular calculi, resulting in lumbar pain, hematuria, recurrent urinary tract infections, and even damage to the renal parenchyma surrounding the diverticulum [2]. Symptomatic calyceal diverticular calculi (CDC) usually require surgical intervention. The main treatments for CDC currently include extracorporeal shock-wave lithotripsy (ESWL), flexible ureterorenoscopy (F-URS), percutaneous nephrolithotomy (PCNL), and laparoscopic surgery. ESWL is a convenient and less-invasive procedure and is often the preferred option for lithotripsy. However, ESWL cannot dilate the narrow diverticular neck, which is not conducive to calculi expulsion [3]. PCNL provides a high stone-free rate for CDC, but it may cause serious complications, such as bleeding, damage to surrounding organs or renal parenchyma, sepsis, and even death, and it is difficult to manage calculi in small diverticula or diverticula located in the upper and ventral calyces [4–6]. Laparoscopy is appropriate for patients with diverticula located on the surface of the renal parenchyma and with thin renal parenchyma, but the operation time of laparoscopy is long, and the surgery is traumatic. It is generally used only for patients who have failed with minimally invasive surgery [7]. F-URS with holmium laser lithotripsy is less-invasive, has fewer complications, and has a fast recovery, but there were only a few clinical reports on F-URS with holmium laser for the treatment of CDC. Therefore, we retrospectively analyzed 27 cases of CDC treated by F-URS with holmium laser in our hospital to explore the efficacy and safety of this technique. This retrospective study was reviewed and approved by our institution's ethics committee, and the patient informed consent was waived.

## Patients and methods

### Patients

From May 2018 to May 2021, 27 patients with symptomatic CDC were admitted to the Department of Urology of the Zigong First People's Hospital and underwent treatment by F-URS with holmium laser. All patients finished their blood routine, blood biochemistry, coagulation function, electrolytes, urinalysis, and urine culture before surgery. For patients with urinary tract infections, antibiotics were used to control the infection, and surgery was performed after the infection was controlled. All patients finished preoperative ultrasonography, abdominal plain film (KUB) + intravenous pyelography (IVP), abdominal computerized tomography (CT), abdominal contrast-enhanced CT, or CT urography (CTU) to identify the size, location, and hardness of calculi.

### Surgical methods

**Fig. 1:**
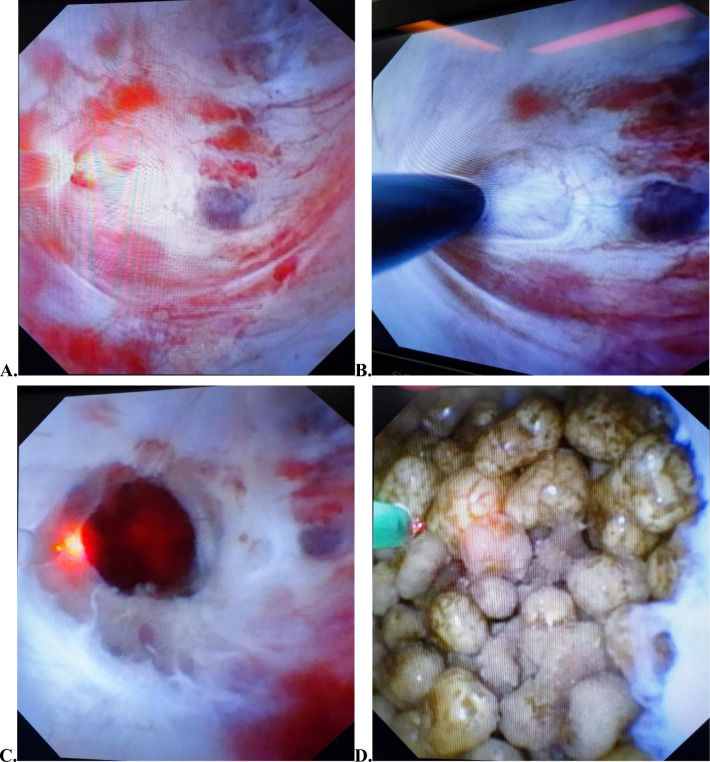
The procedure of F-URS with holmium laser treatment for CDC. **A** The tip of the holmium laser was pointing at the diverticular neck; **B** insertion of a zebra guidewire into the diverticular neck for exploration;** C** holmium laser incision of the diverticular neck opening along the mucosal hypovascular areas; **D** flexible ureteroscope entered the diverticulum for lithotripsy (multiple stones can be seen in the diverticulum)

Under general anesthesia, the patient was positioned in the lithotomy position. The rigid ureteroscope (Wolf F8/9.8) was guided into the renal pelvis with a zebra guidewire (Copper S515035), and the distal end of the zebra guidewire was retained in the pelvis. The flexible ureteroscope sheath (Cook F14) was placed along the zebra guidewire to reach the ureteropelvic junction. The electronic flexible ureteroscope (Olympus URF-V) was placed into the sheath and reached the renal pelvis. Flexible ureteroscope explored the renal pelvis and calyces to find the diverticular neck. If the diverticular neck was difficult to find, 10% methylene blue could be injected into the renal pelvis and kept for about 5 min, and then quickly flush out the methylene blue in the collecting system by saline, and the location with blue cloud-like liquid outflow was the diverticular neck. If the diverticular neck still cannot be found, an intraoperative ultrasonography was used to localize the calculi in real-time. Strong echoes with shadows within the cystic anechoic zone were the area where the stone was located. Explored the plane where the stone was located by flexible ureteroscope under ultrasound guidance and carefully detected any abnormality in the mucosa of the area. After finding the diverticular neck, probed the diverticular neck with the tip of the guidewire and observed the thickness of the mucosa and the distribution of blood vessels at the neck (Fig. 1A, B). A 200 μm holmium laser (Energy to 0.8 J/20 HZ) was used to incise the thin and less-vascularized mucosa of the diverticular neck and enlarged the neck for smooth entry of the flexible ureteroscope (Fig. 1C). The flexible ureteroscope entered the diverticulum and found the stones and the holmium laser (energy to 0.8–1 J/25–30 HZ) fragmented the stones to ≤ 3 mm (Fig. 1D). The patient’s position was adjusted according to the location of the diverticulum and the fragments within the diverticulum were flushed out to the renal pelvis to facilitate drainage. For hard and large fragments, a stone basket was used to withdraw them. When lithotripsy was completed, a zebra guidewire was placed in the renal pelvis or diverticulum, and an F5 double J tube (Urovision ST-205528-UE) was placed in the ureter along the zebra guidewire.

### Postoperative follow-up

The KUB was rechecked on the 2nd postoperative day to assess the position of the double J tube for any abnormality. KUB, ultrasonography, or abdominal CT were performed 1 month after the operation for assessment of stone clearance, and stone-free was defined as no stone fragments residue or residual fragments ≤ 3 mm. Stone fragments > 3 mm were defined as stone residue. Thereafter, ultrasonography or abdominal CT was performed every 3–6 months until 1 year after the operation to assess if there were any recurrence of stones.

### Statistical analysis

IBM SPSS 23.0 statistical software was used for data analysis. The measurement data were expressed as mean ± standard deviation (SD); the count data were expressed as cases (%); the difference between groups was determined by Fisher's exact test. *p* < 0.05 was considered statistically significant.

## Results

The 27 cases in this group were 8 males and 19 females. The mean age was 44.1 ± 14.2 (20–73) years. The mean BMI was 22.8 ± 2.9 (17.9–27.1) kg/m^2^. The calculi were in the upper, middle, and lower renal calyces in 15, 8, and 4 cases, respectively. The mean diameter of calculi was 5.8 ± 3.1 (2–14.4) mm and the mean CT value was 1014 ± 250.8 (572–1491) HU. The mean diameter of diverticula was 16.7 ± 5 (8–27) mm. The patient and stone characteristics are listed in Table 1.

**Table 1:** Patient characteristics

Variable	No	%	Mean ± SD	Minimum–maximum
Age (years)			44.1 ± 14.2	20–73
Gender				
Female	19	70.4%		
Male	8	29.6%		
BMI (kg/m^2^)			22.8 ± 2.9	17.9–27.1
Symptoms				
Renal colic	18	66.7%		
UTI	6	22.2%		
Hematuria	3	11.1%		
Previous SWL	4	14.8%		
With UTI	11	40.7%		
Stone side				
Left	15	55.6%		
Right	12	44.4%		
Stone length (mm)			5.8 ± 3.1	2–14.4
CT value of stone (HU)			1014 ± 250.8	572–1491
Diverticulum Length (mm)			16.7 ± 5	8–27
Stone number				
Single	7	25.9%		
Multiple	20	74.1%		
Stone site				
Upper calyx	15	55.6%		
Middle calyx	8	29.6%		
Lower calyx	4	14.8%		

*SD* standard deviation, *BMI* body mass index, *UTI* urinary tract infection, *SWL* shock-wave lithotripsy, *HU* Hounsfield unit

Diverticula were successfully found in all 27 patients. Lithotripsy was successful in 25 (92.6%) cases and failed in 2 (7.4%) cases. The 2 failed lithotripsy cases were both presented with slender diverticular necks, which could not be incised under F-URS. One case was successfully lithotripsy after conversion to PCNL; another case refused to undergo PCNL and was discharged with relief of lumbar pain after the operation. All the 25 cases with successful lithotripsy by F-URS with holmium laser did not present serious complications intraoperatively, such as ureteral perforation, mucosal avulsion, or hemorrhage. Four patients had postoperative minor complications (Clavien classification I–II), including one case of fever and two cases of bleeding, all of which recovered after treatment with anti-infection or hemostasis. One patient developed a subperitoneal hematoma after the operation and was discharged after the condition was stabilized, and the hematoma disappeared on re-examination 3 months later. The mean operative time of the 25 patients was 76.9 ± 35.5 (43–200) min and the mean hospital stay was 4.4 ± 1.7 (3–12) days. During the 1-month postoperative follow-up, 20 patients had complete stone clearance, with a stone-free rate of 80%. The stone-free rates in the upper, middle, and lower calyces were 85.7%, 71.4%, and 75%, respectively, with no statistically significant difference between the 3 groups (*p* = 0.808) (Table 3). Five patients had stones residual, and two patients were treated with ESWL and had complete stone drainage on review; three patients were chosen for conservative observation. The stone-free rate after second-stage treatment was 88%. The 22 patients with complete stone clearance were followed up for 5.3 ± 2.6 (3–12) months and no stone recurrence was observed. The operative and postoperative outcomes are listed in Table 2.

**Table 2:** Operative and postoperative outcomes

Variable	No (%)	Mean ± SD	Minimum–maximum
Success rate	25(92.6%)		
Operative time (min)		76.9 ± 35.5	43–200
Hospital stays (day)		4.4 ± 1.7	3–12
Complications (Clavien I–II)	4(16%)		
Stone-free rate	20(80%)		
Final treatment stone-free rate	22(88%)		
Follow-up time (month)*		5.3 ± 2.6	3–12

*SD* standard deviation

*The follow-up time specifically refers to the 22 cases in which the stones were completely cleared

## Discussion

Despite the low incidence of CDC, they are relatively tricky to manage. For asymptomatic patients with small diverticular calculi, it can be observed conservatively and followed up regularly; for asymptomatic patients with certain requirements or special occupations (e.g., pilots), surgical treatment is also applicable [8]. When diverticular calculi cause lumbar pain, hematuria, and recurrent urinary tract infections, they should be managed surgically [6].

Currently, the main treatments for CDC include ESWL, F-URS, PCNL, and laparoscopic surgery. ESWL is a convenient and less-invasive procedure, but ESWL cannot dilate the narrow diverticular neck, fragmented stones are difficult to discharge from the diverticulum, and the recurrence rate of stones is high [3]. Turna et al. treated 38 patients with CDC by ESWL with a symptomatic relief rate of 61%, and only 8 patients (21%) had successful stone expulsion at a 3-month follow-up [3]. There was even a case of bacterial sepsis caused by CDC treated with ESWL has been reported in the literature [9]. In both our and previous studies, there were patients who first underwent ESWL but failed treatment and eventually chose F-URS to manage their stones [10, 11]. PCNL and F-URS are currently the most commonly used methods for the management of CDC. Literature reports that PCNL treatment of CDC has a stone clearance rate of 87.5–100%, a diverticulum closure rate of 76–100%, and symptomatic relief in more than 90% of patients at follow-up [6, 12]. In addition, after the removal of diverticular calculi, PCNL can mechanically dilate the diverticular neck to allow adequate drainage, or it can cauterize the mucosa of the diverticular cavity to promote its closure [13]. However, PCNL also has its limitations; for example, when dealing with small diverticula or diverticular calculi located in the upper and ventral calyces, due to the special location and angle of the puncture, it may cause serious complications, such as pleural, intestinal, and great vessel injuries [5, 6, 14]. Laparoscopy is appropriate for patients with diverticula located on the surface of the renal parenchyma and with thin renal parenchyma, but laparoscopic surgery is complicated and traumatic, and it is difficult to deal with diverticular calculi in the deeper part of the renal parenchyma, which restricts its wide application, and it is generally used as a complementary method to minimally invasive surgery [15].

Fuchs et al. made the first report on the use of F-URS for the management of CDC. Although intracavitary holmium laser lithotripsy was not available at that time, they successfully managed 15 cases of CDC with a stone-free rate of 73.3% by F-URS with electrohydraulic lithotripsy and ESWL [16]. In recent years, with the development of F-URS and holmium laser equipment and technology, F-URS with holmium laser has shown unique advantages in the management of CDC. First, F-URS utilizes the natural lumen of the human body, which has the advantages of less trauma, better healing, and faster recovery. Chen et al. reported that F-URS for the treatment of CDC achieved an initial surgical stone-free rate of 81.4% and a symptom relief rate of 90%. The overall stone-free rate was 93% after the second-stage surgery; the complication rate was 11.6%, all of which were minor complications (Clavien classification I–II) [11]. Bas et al. compared the efficacy of F-URS versus PCNL in the management of CDC. It was found that there was no significant difference between the two groups in terms of operation success rate, symptom relief rate, and stone-free rate (*p* = 0.537, *p* = 0.880, *p* = 0.539), but the incidence of major complications (Clavien III) was higher in the PCNL group [6]. Furthermore, the F-URS with holmium laser can incise the narrow diverticular neck, which completely solves the fundamental problem of diverticular neck stenosis and reduces the chance of stone residue and recurrence after surgery [14, 17]. Koopman et al. reported that 94% of patients had their diverticular neck successfully dilated or incised during F-URS treatment of CDC, with a postoperative stone-free rate of 90% [18]. In the current study, 92.6% of the 27 patients had their diverticular necks successfully incised for lithotripsy. Only 4 patients suffered minor complications postoperatively. The stone-free rate was 80% after initial surgery and 88% after second-stage treatment, which was like the results of Chen et al.’s research and reconfirmed the high efficiency and safety of F-URS with holmium laser management of CDC.

**Fig. 2:**
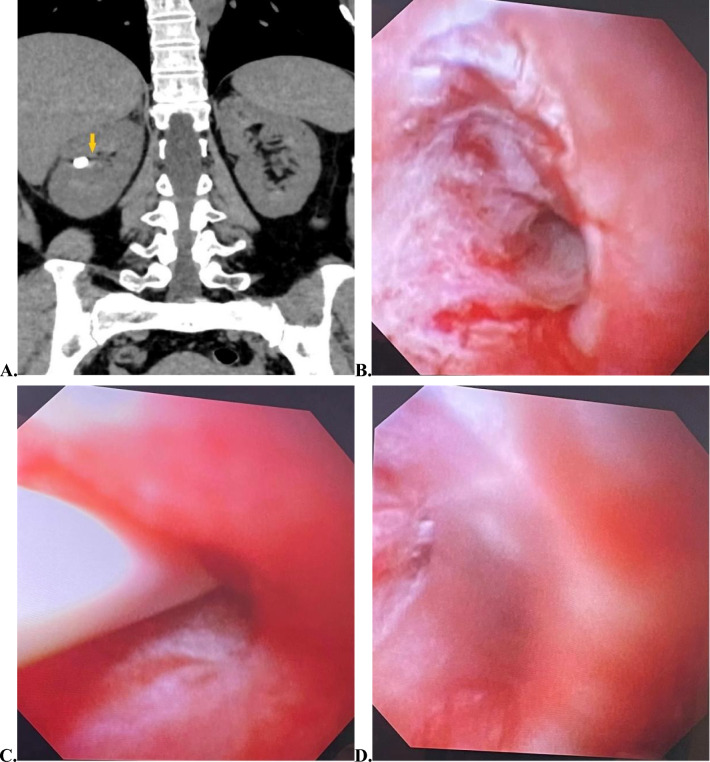
Preoperative and intraoperative imaging data of failed F-URS lithotripsy case 1. **A** CT coronal image shows that the stone was in the diverticulum of the middle calyx of the right kidney. The slender neck of the diverticulum (approximately 6 mm in length) was marked by the yellow arrow; **B** the diverticular neck opening; **C** guidewire exploration of the diverticular neck opening; **D** purulent fluid was seen flowing from the opening after removal of the guidewire

In theory, F-URS can treat all kidney calculi [4]. However, due to the limitations of infundibular pelvic angle (IPA), infundibular length and width of the lower calyx, and curvature of the end of the flexible ureteroscope, it was difficult to handle calculi in some diverticula of the lower calyx, and the stone-free rate was low [14]. Bas et al. found that the success rate of F-URS in the management of diverticular calculi of the lower calyx was only 60% influenced by the angle of deflection [6]. However, Sejiny et al. reported that although the efficacy of F-URS in treating calculi within the lower calyces diverticulum was lower than that of the upper and middle calyces, the difference between the three groups was not statistically significant (*p* = 0.369) [14]. Boonyapalanant et al. also found that the stone-free rate of F-URS treatment for CDC was not affected by the location of the diverticulum but affected by the size of the calculi and the length of the diverticular neck. They found that F-URS treatment of CDC with a stone size < 1.5 cm and a diverticular neck length < 0.4 cm achieved a high rate of stone clearance [13]. Chen et al. concluded that F-URS was appropriate for the management of CDC with large diverticular openings and short necks, but not appropriate for patients with slender diverticular necks or atresia diverticulum [11]. Our findings coincided with Sejiny’s as well as Boonyapalanant’s views. In our group, there were no statistically significant differences found in stone-free rates between the upper, middle, and lower calyx diverticula (*p* = 0.808). The 2 surgery failure cases both had slender diverticular necks, thick peri-neck renal parenchyma, and abundant blood supply. Concerned about bleeding after holmium laser incision, flexible ureteroscopic lithotripsy was not performed. Case 1 patient was admitted with lumbar pain, and the calculi were in the diverticulum of the right upper calyx with a diverticular neck length of about 6 mm. During the operation, a guide wire was used to probe the diverticular neck, and purulent urine was found to flow from the diverticular neck, but the patient refused to undergo PCNL. After the operation, the patient's lumbar pain was relieved (we considered that the patient's lumbar pain was relieved owing to the release of the diverticular neck obstruction during intraoperative guidewire exploration) (Fig. 2A–D), and he was discharged from our hospital. In another case, the calculi were in the middle calyx of the left kidney, and the diverticulum was found to have a small opening with a slender neck, so the ureteroscope could not enter the diverticulum, and the stone was successfully fragmented after PCNL.

The key to F-URS treatment of CDC is the successful finding of the diverticular opening. We summarized several experiences: ① Preoperative ultrasonography, KUB + IVP, CT, CTU, and other relevant imaging examinations should be completed to identify the relationship between the diverticulum and the collecting system; ② F-URS should be performed as gently as possible to avoid intraoperative bleeding affecting the vision; ③ Maintain moderate perfusion pressure. In some cases with needle-like diverticular openings, an excessively low perfusion pressure makes it difficult to dilate the opening; an excessively high perfusion pressure increases the risk of urogenic sepsis; ④ For the suspected diverticular opening, insert a guidewire to explore, some cases can observe purulent urine flowing around the guidewire after insertion, and this sign can help us confirm that this is the diverticular opening; ⑤ If the diverticular opening cannot be found, try a methylene blue examination to look for it or look for it under ultrasound guidance. In addition, a successful and safe diverticular neck incision also appears to be extremely necessary. The following points should be noted during the incision process: ① When Holmium laser incises the diverticular neck, the energy parameter setting at low energy and high frequency (0.8–1.0 J/20–30 HZ) is recommended to have a better incision and hemostasis effect; ② The neck incision should be made along the thin mucosa of the diverticulum as much as possible and should be stopped when adjacent to thicker renal parenchymal tissue to avoid bleeding. The use of narrow band imaging (NBI) mode intraoperatively helps to identify the vascular distribution while not affecting the cutting procedure; ③ Under the premise of no bleeding, the diverticular neck incision should be large enough to enable the diverticulum to drain smoothly and prevent the stone recurrence after surgery; ④ Fragments within the diverticulum should be flushed out to the renal pelvis or adjacent calyces as far as possible to facilitate stone drainage; for lower calyces’ fragments, the Trendelenburg position can be used for flushing during the operation.

As for the management of CDC by F-URS, we believe that there are still many issues that need to be addressed. At first, most scholars believed that the diverticular lining should be fulgurated when CDC is treated with PCNL, resulting in collapse and closure of the diverticular cavity to prevent the recurrence of stone [12, 19]. Kim et al. reported that after intraoperative fulgurate through PCNL, all diverticula were reduced in size and 87.5% of diverticula disappeared at 3 months of follow-up [19]. Several existing studies have reported a high rate of stone-free during F-URS for the management of CDC but did not include factors such as whether the stones recurred after the operation and whether the diverticulum was closed [10, 11, 14]. Compared to PCNL, F-URS with holmium laser can sufficiently incise and drain the diverticular neck; therefore, whether it is necessary to close the diverticulum by fulguration needs further study. In addition, there is no consensus on whether the proximal end of the double J tube needs to be placed in the diverticulum after lithotripsy. Boonyapalanant et al. suggested that placing a double J tube into the renal calyces can provide drainage support, facilitate stone removal, and prevent restenosis of the diverticular neck [13]. Of the 25 patients successfully lithotripsy under F-URS in this study, 9 cases had the proximal end of the double J tube placed in the renal pelvis and 16 cases had the proximal end of the double J tube placed in the diverticulum, and no statistically significant difference was found in the stone-free rate comparing the two groups (*p* = 0.312) (Table 3). It can be seen that the placement of the double J tube does not affect the stone-free rate as long as the diverticular neck is adequately incised by the F-URS with holmium laser. Unfortunately, we did not follow up on the recurrence of diverticular neck stenosis. It is certain that when placing the guidewire, the position and depth of the tip of the guidewire must be clearly defined under the direct view of the flexible ureteroscope, thus avoiding the tip of the guidewire to penetrate the renal parenchyma, which may lead to double J tube ectopia. In the case of a postoperative subperitoneal hematoma in our group, the guidewire was not placed under direct view of the flexible ureteroscope, and the postoperative examination revealed that the double J tube penetrated the renal parenchyma in the diverticular dome, with the tip located under the peritoneum, and caused a subperitoneal hematoma. Due to the special anatomical location of the diverticula, most of the renal parenchyma in the diverticular dome is thin, which increases the probability of guidewire penetration into the renal parenchyma, and this case also provides experience for our following work.

**Table 3:** Association of different variables and stone-free rate (*n* = 25)

Variable	Treatment success	Treatment failure	*P*	Test
Stone-free rate (calyceal diverticulum Location)				
Upper calyx	12(85.7%)	2(14.3%)	0.808	Fisher’s exact test
Middle calyx	5(71.4%)	2(28.6%)		
Lower calyx	3(75%)	1(25%)		
Stone-free rate (double J tube position)				
Renal pelvis	6(66.7%)	3(33.3%)	0.312	Fisher’s exact test
Calyceal diverticula	14(87.5%)	2(12.5%)		

## Conclusion

In conclusion, F-URS with holmium laser is a safe and effective choice for the treatment of CDC, because it utilizes the natural lumen of the body and has the advantages of less trauma, fewer complications, and higher stone-free rates. However, there are still many limitations in this study. First, we did not conduct a stone composition analysis, thus failing to guide the patient for better prevention of stone recurrence and further treatment. Second, since there were too few cases of CDC in the lower calyces in this study (only 4 cases), we were unable to analyze whether the treatment of CDC in the lower calyces by F-URS was affected by the anatomical parameters of the lower calyces, such as the IPA, infundibular width, and infundibular length. Furthermore, this study was a single-center, retrospective study with a small sample size and there were no control groups. A large sample, prospective randomized-controlled study is still needed for further validation.

## Data Availability

The data supporting the results of this study are available from the corresponding author upon reasonable request.
